# Expression of Oncofetal Antigen 5T4 in Murine Taste Papillae

**DOI:** 10.3389/fncel.2019.00343

**Published:** 2019-07-31

**Authors:** Yuka Takahashi, Hiroo Takahashi, Peter L. Stern, Tadaaki Kirita, Akio Tsuboi

**Affiliations:** ^1^Department of Oral and Maxillofacial Surgery, Nara Medical University, Kashihara, Japan; ^2^Department of Molecular Neurobiology, Faculty of Medicine, Kagawa University, Miki-cho, Japan; ^3^Division of Molecular and Clinical Cancer Sciences, University of Manchester, Manchester, United Kingdom; ^4^Laboratory for Cellular and Molecular Neurobiology, Graduate School of Frontier Biosciences, Osaka University, Suita, Japan

**Keywords:** 5T4 oncofetal antigen, circumvallate papilla, taste bud, epithelial progenitor, taste precursor cell, taste receptor cell, mouse

## Abstract

**Background:** Multicellular taste buds located within taste papillae on the tongue mediate taste sensation. In taste papillae, taste bud cells (TBCs), such as taste receptor cells and taste precursor cells, and the surrounding lingual epithelium including epithelial progenitors (also called taste stem/progenitor cells) are maintained by continuous cell turnover throughout life. However, it remains unknown how the cells constituting taste buds proliferate and differentiate to maintain taste bud tissue. Based on *in situ* hybridization (ISH) screening, we demonstrated that the oncofetal antigen *5T4* (also known as trophoblast glycoprotein: *TPBG*) gene is expressed in the adult mouse tongue.

**Results:** In immunohistochemistry of coronal tongue sections, 5T4 protein was detected at a low level exclusively in the basal part of the lingual epithelium in developing and adult mice, and at a high level particularly in foliate papillae and circumvallate papillae (CVPs). Furthermore, immunohistochemistry of the basal part of CVPs indicated that the proliferation marker PCNA (proliferating cell nuclear antigen) co-localized with 5T4. *5T4* was strongly expressed in Krt5^+^ epithelial progenitors and Shh^+^ taste precursor cells, but weakly in mature taste receptor cells. The number of proliferating cells in the CVP was higher in *5T4-*knockout mice than in wild-type (WT) mice, while neither cell differentiation nor the size of taste buds differed between these two groups of mice. Notably, X-ray irradiation enhanced cell proliferation more in *5T4*-knockout mice than in WT mice.

**Conclusion:** Our results suggest that 5T4, expressed in epithelial progenitors (taste stem/progenitor cells), and taste precursor cells, may influence the maintenance of taste papillae under both normal and injury conditions.

## Introduction

Taste buds comprise taste bud cells (TBCs), such as taste receptor cells and taste precursor cells, and are surrounded by the lingual epithelium, which includes epithelial progenitors (taste stem/progenitor cells). Each taste bud contains approximately 50–100 TBCs, which are classified into four types called I, II, III, and IV (Feng et al., 2014; Barlow, 2015; Yang et al., 2019). Type I, II, and III TBCs are mature taste receptor cells and involved in taste perception, whereas Type IV TBCs are immature taste precursor cells. Type IV TBCs in the basal part of taste buds express *Shh* (Miura et al., 2001, 2006; Takeda et al., 2013) and mature into Type I, II, and III taste receptor cells, in proportion reflecting cell type ratios in taste buds (Type I > II > III) (Miura et al., 2014). TBCs and neighboring epithelial cells differentiate from epithelial progenitor cells (also called taste stem/progenitor cells) expressing *Krt5* and *Krt14*, which are located in the basal part of the lingual epithelium outside the taste bud (Okubo et al., 2009; Ohmoto et al., 2017). These cells are continuously turned over every 10–14 days throughout life (Beidler and Smallman, 1965; Farbman, 1980; Perea-Martinez et al., 2013). The Wnt/ß-catenin pathway is known to play a key regulatory role in taste bud homeostasis (Barlow, 2015). Furthermore, Wnt/ß-catenin signaling is necessary and sufficient for induction of taste placodes during development (Iwatsuki et al., 2007; Liu et al., 2007). Overexpression of *β-catenin* in Krt5^+^ epithelial progenitors facilitates the generation of TBCs and inhibits the production of neighboring epithelial cells (Gaillard et al., 2015). By contrast, knockdown of *β-catenin* in epithelial progenitors leads to rapid depletion of Krt5^+^ progenitors and Shh^+^ precursors in the fungiform papilla and CVP (Gaillard et al., 2017). Wnt/β-catenin signaling also controls differentiation into each type of TBC (Gaillard et al., 2015).

However, it remains unknown how TBCs proliferate and differentiate to maintain taste bud tissue. We searched for mouse genes that are expressed in the basal part of taste buds and found that a gene encoding a single-pass transmembrane glycoprotein, termed oncofetal antigen *5T4* (also known as trophoblast glycoprotein: *TPBG*), is significantly expressed. *5T4* is expressed not only in various types of human cancers, including those of the lung, esophagus, stomach, pancreas, bladder, uterus, ovary, and testis (Southall et al., 1990), but also in normal tissues such as trophoblasts in the placenta and several regions of the brain (King et al., 1999; Imamura et al., 2006), including those mentioned in our previous studies (Yoshihara et al., 2012, 2016; Takahashi et al., 2016, 2018). *5T4* regulates the early differentiation and CXCR4-mediated chemotaxis of embryonic stem cells (Ward et al., 2003; Southgate et al., 2010). During mouse embryogenesis, *5T4* is expressed in *Krt14^+^* epithelial progenitor cells in the vibrissae hair follicle and epidermis (Barrow et al., 2005). *5T4* reduces the proliferation of the murine A9 cell line (Carsberg et al., 1995) and embryonic stem cells (Ward et al., 2003). *5T4* is also associated with mobilization of cancer stem cells (Harrop et al., 2019). These studies suggest that *5T4* is multifunctional and influences cell proliferation, differentiation and migration during development, and cancer progression. Furthermore, 5T4 negatively regulates the canonical Wnt/β-catenin pathway (Kagermeier-Schenk et al., 2011; Zhao et al., 2014; Yoshihara et al., 2016) that controls the development and maintenance of taste buds (Barlow, 2015). However, no study has investigated how and why *5T4* is expressed in the mouse tongue. Therefore, we performed detailed immunohistochemical analyses to elucidate the potential role of 5T4 in TBC differentiation and progenitor proliferation.

## Materials and Methods

### Animals

Animal experiments were approved by the Animal Care Committee of Nara Medical University in accordance with the policies established in the NIH Guide for the Care and Use of Laboratory Animals. Male and female C57BL/6 mice were purchased from Japan SLC (Shizuoka, Japan). A *5T4-*knockout (KO) mouse line was generated as described previously (Southgate et al., 2010). *5T4* heterozygous mice (*5T4^+/-^*) were backcrossed over 15 generations with mice of the C57BL/6 wild-type (WT) background. *5T4*-KO mice were compared with their WT littermates, which were used as controls. To prepare tongue samples, mice were anesthetized with sodium pentobarbital intraperitoneally (100 μg/g of body weight), and perfused with 4% paraformaldehyde (PFA) in phosphate-buffered saline (PBS), following post-fixation for 10 min. When analyzing tongue areas, images of fixed tongues were acquired from the dorsal side by using an SZX16 stereomicroscope (Olympus, Tokyo, Japan) equipped with a CCD camera (DP22; Olympus). The tongue area was defined as that extending from the tongue tip to the tongue base and was measured in dorsal images using Image-J software (National Institutes of Health, MD, United States).

### Immunohistochemistry (IHC)

PFA-fixed tongues were placed in 30% sucrose, and embedded in OCT compound (Sakura Finetek, Tokyo, Japan) on liquid nitrogen. Coronal sections (12 μm) were cut with a CM3000 Cryostat (Leica, Nussloch, Germany) and collected on MAS-coated slide glasses (Matsunami, Osaka, Japan). To retrieve the antigen, sections were pretreated in citrate buffer (10 mM, pH 6.0) at 65°C for 3 h before IHC, except in the case of NCAM staining (Figure 4A). IHC of mouse tongue sections was performed as previously described (Yoshihara et al., 2005, 2012; Takahashi et al., 2016). The following antibodies were used: sheep anti-5T4 (1:1000; R&D Systems, MN, United States), rabbit anti-5T4 (1:1000; Imamura et al., 2006), goat anti-Cldn7 (1:1000; Santa Cruz Biotechnology, TX, United States), rabbit anti-Cldn7 (1:1000; Abcam, Cambridge, United Kingdom), mouse anti-proliferating cell nuclear antigen (PCNA; 1:1000; Santa Cruz Biotechnology), rabbit anti-Krt5 (1:1000; BioLegend, CA, United States), goat anti-Shh (1:1000; R&D Systems), rat anti-5-bromo-2-deoxyuridine (BrdU; 1:1000; Abcam), Gustducin (1:1000; Santa Cruz Biotechnology, TX, United States), NCAM (1:1000; Merck Millipore, MA, United States), and rabbit anti-GFP (1:1000; Thermo Fisher Scientific, MA, United States). DyLight 488-, DyLight 549-, and DyLight 649-conjugated secondary antibodies were purchased from Jackson ImmunoResearch (West Grove, PA, United States). Counterstaining of nuclei with DAPI was performed on all sections. Images were acquired using an IX71 microscope (Olympus, Tokyo, Japan) equipped with a CCD camera (DP30BW; Olympus) or with a confocal laser microscope (FV1000-D; Olympus). The Cldn7-positive area was defined as the taste bud. For quantification, the number of taste buds (Cldn7^+^) within the CVP was counted in Z-stack images acquired with FV1000-D.

### *In situ* Hybridization (ISH)

Mice were anesthetized with sodium pentobarbital intraperitoneally (100 μg/g of body weight), and perfused with PBS. Non-fixed tongues were embedded in OCT compound (Sakura Finetek) on liquid nitrogen. Coronal sections (12 μm) were cut with a CM3000 Cryostat (Leica) and collected on MAS-coated slide glasses (Matsunami). ISH was performed as described previously (Oike et al., 2006). The coding regions of *5T4* (434-1714 nt; GenBenk No. NM_001164792.1) and *Tas1r2* (71-1036 nt; GenBenk no. NM_031873.1) were used as templates for the synthesis of digoxigenin-labeled RNA probes (Roche Diagnostics, Basel, Switzerland).

### BrdU Labeling and Detection

To determine the number of newly generated cells, BrdU (10 mg/ml; Nacalai Tesque, Kyoto, Japan), a marker of cell proliferation, was administered intraperitoneally (100 μg/g of body weight dissolved in PBS). Six hours after injection, BrdU-labeled cells were detected as described previously (Takahashi et al., 2016). For quantification, the number of BrdU^+^ cells within the CVP was measured in Z-stack images acquired with a confocal laser microscope (FV1000-D). Signals larger than 10 μm^2^ were counted as BrdU^+^ nuclei. In the same images, the CVP area was measured using Olympus Fluoview software (Olympus), and the density of BrdU^+^ cells was calculated.

### Quantitative PCR (qPCR) Analysis

Wild-type and *5T4*-KO mice aged 6 weeks (postnatal day 42: P42) were anesthetized with sodium pentobarbital intraperitoneally (100 μg/g of body weight). Each part of the tongue epithelium (anterior, middle, and CVPs) was manually excised using a scalpel under a zoom stereomicroscope (SZ61; Olympus). mRNA was prepared from three animals of each strain using an miRNeasy Mini Kit (QIAGEN, Hilden Germany) and then cDNA was synthesized using 0.4 μg of each RNA and PrimeScript RT Master Mix (TaKaRa Bio, Shiga, Japan) according to the manufacturers’ protocols. qPCR analysis was performed with PowerUp SYBR Green Master Mix (Thermo Fisher Scientific) on a StepOne Plus System (Thermo Fisher Scientific). The following primers were used: *Actb* forward 5′-catggcattgttaccaactg-3′ and reverse 5′-gtctcaaacatgatctgggtc-3′, *Shh* forward 5′-ccccaattacaaccccgaca-3′ and reverse 5′-acttgtctttgcacctctgagt-3′, *Cldn7* forward 5′-caagggcccgcatactttct-3′ and reverse 5′-tggttccagacaaaagcggt-3′, *Entpd2* forward 5′-gccctcaagtatggcatcgt-3′ and reverse 5′-cgaacatcgcaagagctgtg-3′, *Plcb2* forward 5′-atggagttcctggatgtcacg-3′ and reverse 5′-cggagtttctggctcttggg-3′, *Snap25* forward 5′-caaggcgaacaactggaacg-3′ and reverse 5′-acacacaaagcccgcagaat-3′ and *18S* forward 5′-gtaacccgttgaaccccatt-3′ and reverse 5′-ccatccaatcggtagtagcg-3′. The ΔΔCT method (Livak and Schmittgen, 2001) was used to calculate the relative amounts of mRNA expressed in the CVP using *Actb* mRNA as an internal control, except for the mRNA amounts reported in Supplementary Figure S2B where *18S ribosomal* RNA was used as an internal control.

### Plasmid Construction and Cell Growth Assay

The p*CAG*-*EGFP* vector was constructed by replacing the *CMV* promoter in p*EGFP*-N1 (Takara Bio) with the *CAG* promoter derived from pCL20c-*CAG*-*EGFP* (Takayama et al., 2008). Thereafter, the *EGFP* gene was removed to construct the p*CAG* vector. To construct p*CAG*-*H2B-EGFP* and p*CAG*-*5T4*, the mouse *H2B* and *5T4* genes were cloned using olfactory bulb cDNA. After confirming their sequences, the *H2B* and *5T4* genes were subcloned into the p*CAG-EGFP*, and p*CAG* vectors, respectively.

Human embryonic kidney cells (HEK293T) were purchased from RIKEN Cell Bank (Tsukuba, Japan) and maintained in DMEM (Nacalai Tesque) containing 10% fetal calf serum (Sigma-Aldrich, MO, United States) and penicillin-streptomycin (Nacalai Tesque). When cells in 100 mm dishes reached 70–80% confluency, they were transfected with a mixture of plasmids (10 μg of each), which comprised (A) p*CAG*-*H2B-EGFP* and p*CAG* or (B) p*CAG*-*H2B-EGFP* and p*CAG*-*5T4*, using Lipofectoamine 2000 reagent (Thermo Fisher Scientific) according to the manufacturer’s protocol. Twelve hours after transfection, cells were dispersed with 0.25% trypsin/0.1% EDTA (Nacalai Tesque) and seeded into a 6-well plate at a density of 2.0 × 10^6^ cells per well. The number of EGFP^+^ cells was counted at Day 0, 1, and 2 (Day 0 was 6 h after plating) using a BZ-X710 microscope equipped with an automatic cell counter (KEYENCE, Osaka, Japan).

### Irradiation

Wild-type and *5T4*-KO mice aged 8–10 weeks were anesthetized with chloral hydrate intraperitoneally (350 μg/g of body weight). To limit the irradiation area, anesthetized mice were placed in polypropylene tubes, covered with lead plates except for the anterior part of their body (2 cm), and kept in a handmade holder to maintain the same position in the irradiation device (MBR-1520R; HITACHI, Tokyo, Japan). The area in front of the ear was irradiated with 18.5 Gy of X-rays from the dorsal side. At 10, 16, and 19 days after irradiation, mice were anesthetized with sodium pentobarbital intraperitoneally (100 μg/g of body weight) and perfused with 4% PFA prepared in PBS. IHC of mouse tongue sections was performed as previously described (Yoshihara et al., 2005, 2012; Takahashi et al., 2016).

## Results

### Production of 5T4 in the Basal Part of the Lingual Epithelium in CVPs

In taste buds, TBCs, such as taste receptor cells and taste precursor cells, and the surrounding lingual epithelium are maintained by continuous turnover of cells throughout life. However, it remains unknown how TBCs proliferate and differentiate to maintain taste papillae. Based on *in situ* hybridization (ISH) screening, we demonstrated that the oncofetal antigen gene *5T4* is expressed in the mouse tongue, especially around taste buds (Supplementary Figure S1). Therefore, we performed IHC of tongue sections using an anti-5T4 antibody to examine which type of cells produce 5T4 protein.

**FIGURE 1:**
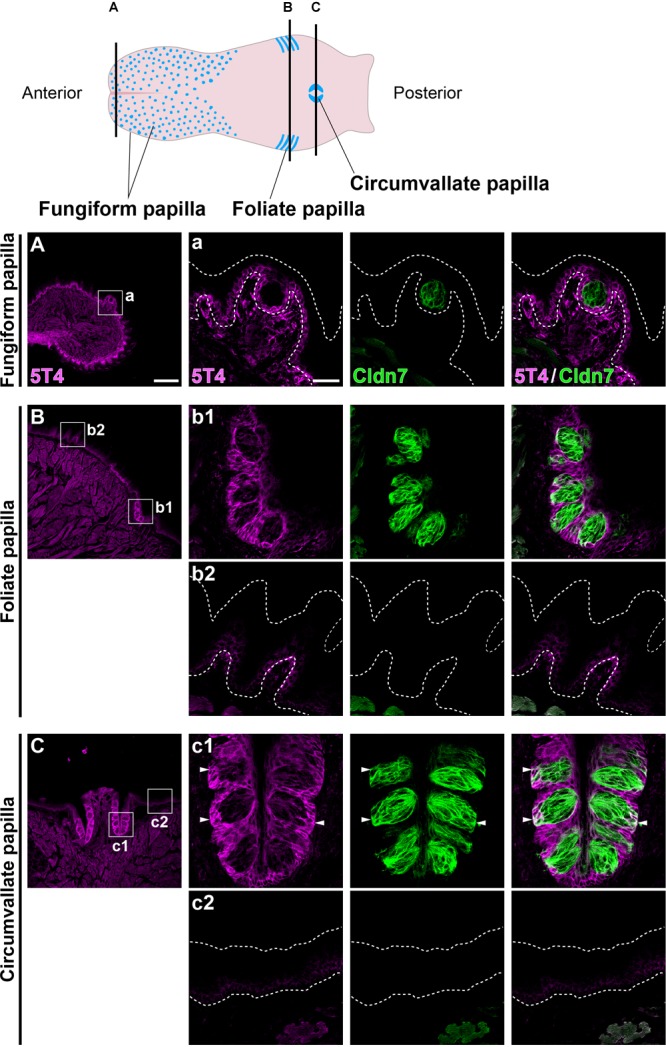
5T4 is abundantly produced within the foliate papilla and CVP. (Top) A schematic drawing of the mouse tongue as modified from Barlow (2015), showing the locations at which coronal sections **(A–C)** were prepared using a cryostat (Bottom). **(A–C)** IHC of the lingual epithelium, including the fungiform papilla **(A)**, foliate papilla **(B)**, and CVP **(C)**, of 6-week-old WT mice using antibodies against 5T4 (magenta) and Cldn7 (a marker of TBCs; green). Enlarged images of the regions enclosed by white squares in **(A–C)** are shown in (a, b1, b2, c1, and c2). All images represent single confocal optical sections [4.51 μm/slice in **(A–C)** and 0.56 μm/slice in (a, b1, b2, c1, and c2)]. Scale bars, 200 μm in **(A)** and 30 μm in (a). White dotted lines indicate the area of the lingual epithelium.

Coronal tongue sections from the anterior to posterior were prepared and subjected to IHC with antibodies against 5T4 and Cldn7 (a marker of TBCs) (Michlig et al., 2007). 5T4 signals were low in the basal part of the entire lingual epithelium, including the filiform and fungiform papillae (Figure 1A–C). 5T4 was strongly detected in the lingual epithelium within the foliate papilla and CVP (Figure 1B,C). In both types of papillae, *5T4* was very weakly expressed in Cldn7^+^ TBCs within taste buds and strongly expressed in surrounding Cldn7^-^ epithelium (Figure 1B,C). 5T4 signals were clearly observed only in the basal portion of taste buds. These results revealed that *5T4* is extensively expressed in the basal part of the entire lingual epithelium and is strongly expressed within the foliate papilla and CVP, including the basal portion of taste buds. 5T4 signals in the connective tissue under the lingual epithelium (Figure 1C) were observed at the same background level in both WT and *5T4*-KO CVPs by IHC analysis (Supplementary Figure S1).

### 5T4 Is Highly Expressed in Proliferating Cells in the Basal Part of the Lingual Epithelium in the CVP

**FIGURE 2:**
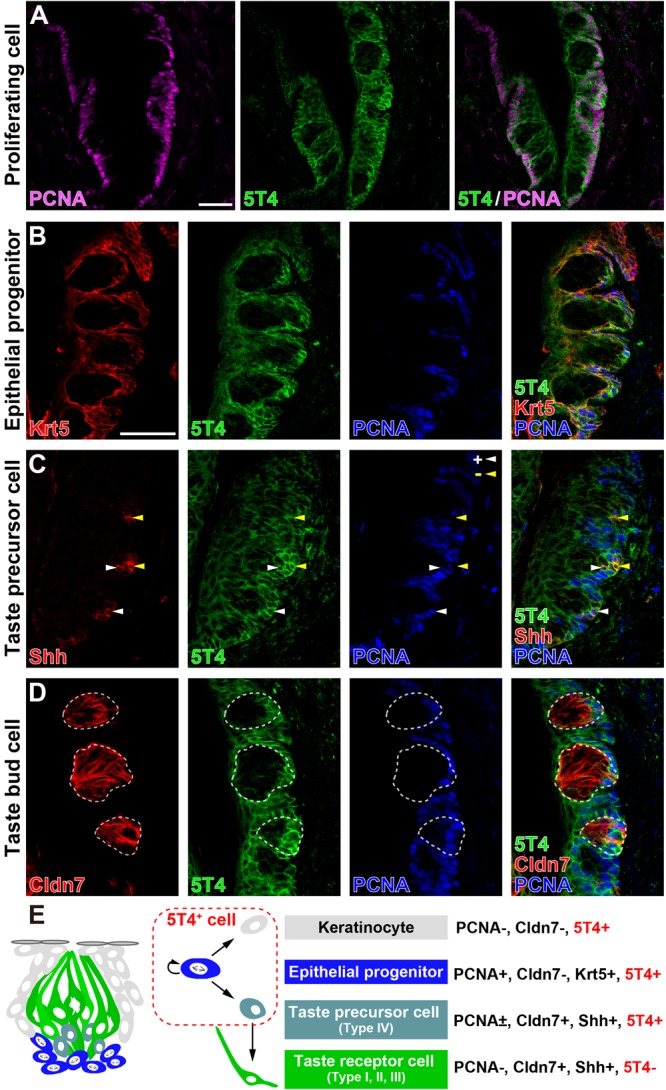
5T4 is highly expressed in proliferating cells in the basal part of the CVP. **(A)** IHC of the CVP using antibodies against 5T4 (green) and PCNA (a marker of proliferating cells; magenta). Scale bars, 50 μm. **(B–D)** IHC of the CVP using antibodies against 5T4 (green) and PCNA (a marker of proliferating cells; blue) in addition to antibodies (red) against various cell type markers, including Krt5 (a marker of epithelial progenitors; **B**), Shh (a marker of taste precursor cells; **C**), and Cldn7 (a marker of TBCs; **D**). PCNA^+^ and PCNA^-^ cells among Shh^+^ taste precursor cells are indicated by white and yellow arrowheads, respectively **(C)**. Scale bars, 50 μm. Each white dotted line indicates the Cldn7^+^ area as a taste bud. All images are single confocal optical sections (0.56 μm/slice). **(E)** A schematic drawing of the mouse taste bud as modified from Barlow (2015), summarizing the results of IHC analysis of epithelial progenitors, taste precursor cells, and taste receptor cells.

Next, to investigate which cells produce 5T4 protein in taste buds within the CVP, coronal tongue sections were prepared and subjected to IHC with antibodies against 5T4 and either PCNA (a marker of proliferating cells), Krt5 (a marker of epithelial progenitors; Okubo et al., 2009), Shh (a marker of taste precursor cells; Miura et al., 2006), or Cldn7 (a marker of TBCs; Michlig et al., 2007). Remarkably, 5T4 signals co-localized with PCNA signals in the basal part inside and outside of taste buds in the CVP (Figure 2A). Triple IHC showed that 5T4 and PCNA signals extensively co-localized with Krt5^+^ epithelial progenitors in the basal part of the lingual epithelium (Figure 2B). These results revealed that 5T4 is highly expressed in proliferating epithelial progenitors (taste stem/progenitor cells) in the basal part of the lingual epithelium. In the taste bud (Cldn7^+^), 5T4- and PCNA-double-positive cells were only located in the basal part (Figure 1B,C, 2D). Taste precursor cells (Type IV TBCs) in the basal part of taste buds differentiate from Krt5^+^ epithelial progenitors (Okubo et al., 2009) and start to express *Shh* at 12 h after their final mitosis (Miura et al., 2006, 2001). Consistently, there were PCNA^+^ and PCNA^-^ populations of Shh^+^ taste precursor cells (Shh^+^ PCNA^-^ = 42.4 ± 6.0%; Figure 2C). 5T4 signals co-localized with both populations of Shh^+^ precursor cells. 5T4 signals were remarkably weak in most portions of taste buds except for the basal part (Figure 2D). These results suggest that *5T4* expression is high in epithelial progenitors and taste precursor cells, but reduced after these cells mature into taste receptor cells. By contrast, 5T4 signals were strong in the entire lingual epithelium outside taste buds including the apical area within the CVP, suggesting that *5T4* expression is sustained until epithelial progenitors differentiate into mature keratinocytes (Cldn7^-^ PCNA^-^) (Figure 2C).

### The Sizes of the Tongue and CVP Do Not Differ Between WT and 5T4-KO Mice

**FIGURE 3:**
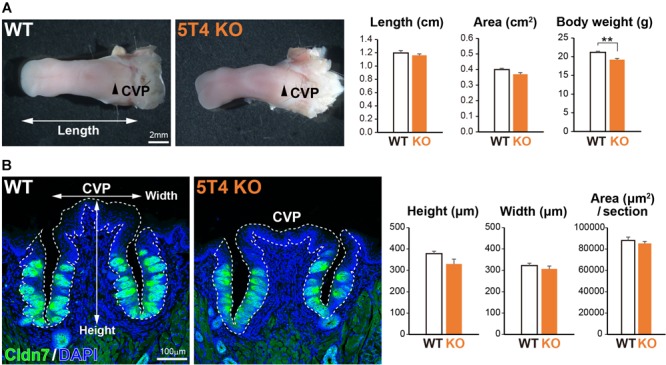
Loss of *5T4* function does not affect the size of the CVP. (**A**, Left) Photographs of tongues prepared from 6-week-old WT and *5T4-*KO mice. CVPs are indicated by arrowheads. Scale bars, 2 mm. (Right) Body weights of 6-week-old WT and *5T4-*KO mice are expressed as the mean ± SEM (^∗∗^*p* < 0.01, Student’s *t* test; *n* = 6 WT mice and *n* = 5 *5T4*-KO mice). The lengths and areas of tongues prepared from 6-week-old WT and *5T4*-KO mice are expressed as the mean ± SEM. The tongue area was defined as that ranging from the tongue tip to the tongue base. (**B**, Left) IHC of the CVP from WT and *5T4*-KO mice with an anti-Cldn7 antibody (a marker of TBCs; green) and DAPI (blue). Scale bars, 100 μm. (Right) Average heights, widths, and areas of the CVP are expressed as the mean ± SEM (*n* = 6 sections per bar from three mice). Images represent Z-stack projections of three sections (2.20 μm/slice).

Next, to investigate how KO of *5T4* affects the lingual epithelium, we compared *5T4*-KO and WT mice. *5T4*-KO mice were born normally and had no obvious abnormalities in mating, nursing, or feeding behavior, although they exhibited olfactory dysfunction (Takahashi et al., 2016, 2018). The body weight of *5T4*-KO mice tended to be lower than that of WT mice (WT, 21.2 ± 0.3 g; *5T4-*KO, 19.2 ± 0.3 g; Figure 3A). However, the size of the tongue did not differ between WT and *5T4*-KO mice (Figure 3A). In addition, we performed IHC with an anti-Cldn7 antibody to investigate the size and morphology of the CVP. The height and width of the CVP did not significantly differ between WT and *5T4-*KO mice. Cldn7^+^ TBCs were similarly, detected in *5T4-*KO and WT mice (Figure 3B).

### Differentiation of Taste Receptor Cells Does Not Differ Between *5T4-*KO and WT Mice

**FIGURE 4:**
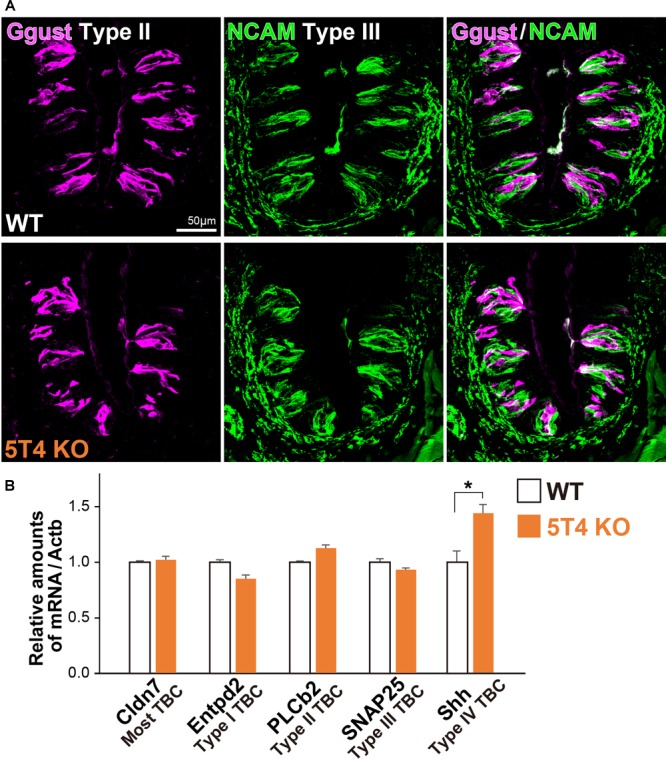
Differentiation of taste receptor cells does not differ between *5T4*-KO and WT mice. **(A)** IHC of the CVPs from WT and *5T4-*KO mice with antibodies against Ggust (a marker of Type II TBCs; magenta) and NCAM (a marker of Type III TBCs; green). Scale bars, 50 μm. Images are Z-stack projections of seven sections (0.64 μm/slice). **(B)** RT-qPCR analysis of total RNA extracted from the CVPs of WT and *5T4*-KO mice. The level of *Actb* mRNA was used as an internal control (^∗^*p* = 0.038 between WT and *5T4*-KO mice, Student’s *t* test with Bonferroni correction; *n* = 4 per bar). Note that differentiation of taste receptor cells does not differ between *5T4*-KO and WT mice, but *Shh* expression (a marker of taste precursor cells) is higher in *5T4*-KO mice than in WT mice.

Taste bud cells are classified into four types: Type I, II, and III, which are elongate cells, and Type IV, which are situated in the basal part (Feng et al., 2014; Barlow, 2015; Yang et al., 2019). Next, to investigate if KO of *5T4* affects the differentiation of taste receptor cells within the CVP, we performed IHC with antibodies against Ggust (a marker of Type II TBCs) and NCAM (a marker of Type III TBCs). Type II and III TBCs did not appear to differ in differentiation between WT and *5T4*-KO mice (Figure 4A). To further investigate if loss of *5T4* function affects cell differentiation, we performed RT-qPCR analyses using total RNA extracted from CVPs of WT and *5T4*-KO mice. Relative amounts of mRNA expressed in the CVP were calculated using *Actb* mRNA as an internal control. Consistent with the results presented in Figure 4A, relative mRNA expression of markers of Type I (*Entpd2*), II (*PLCb2*), and III (*SNAP25*) TBCs did not significantly differ between WT and *5T4*-KO mice (Figure 4B). These results indicate that loss of *5T4* function does not affect the differentiation of taste precursor cells into mature TBCs in the CVP. By contrast, the relative amount of *Shh* mRNA was higher in *5T4-*KO mice than in WT mice (51.4 ± 5.3% higher in *5T4-*KO mice; Figure 4B). This suggests that the number of taste precursor cells is higher in *5T4*-KO mice than in WT mice. Similar results were obtained (Supplementary Figure S2) when *18S ribosomal* RNA was used as the internal control.

### The Number of Proliferating Cells in the Basal Part of Taste Buds in the CVP Is Significantly Higher in *5T4-*KO Mice Than in WT Mice

**FIGURE 5:**
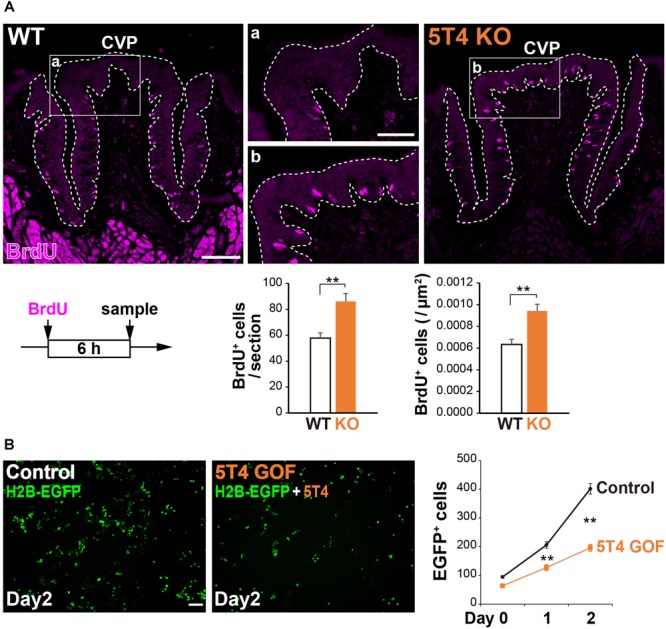
Loss of *5T4* function significantly increases the number of proliferating cells in the CVP. (**A**, Top) Cell proliferation in the WT and *5T4*-KO CVPs. WT and *5T4*-KO mice were intraperitoneally injected with BrdU. Six hours after BrdU injection, cells in the CVP were immunostained with an anti-BrdU antibody (magenta). Scale bars, 100 μm. Images are Z-stack projections of seven sections (0.56 μm/slice). (Bottom) Numbers of BrdU^+^ cells are expressed as the mean ± SEM (^∗∗^*p* < 0.01, Student’s *t* test; *n* = 16 sections from six WT mice, *n* = 8 sections from three *5T4*-KO mice). (**B**, Left) GOF experiments for *5T4* in HEK293T cells. HEK293T cells were transfected with p*CAG*-*H2B-EGFP* alone (Control) or together with p*CAG*-*5T4* (5T4 GOF). One day after transfection, cells were passaged (Day 0) and then cultured for 2 days (Day 2). Scale bars, 100 μm. (Right) Numbers of EGFP^+^ cells at Day 0–2 are expressed as the mean ± SEM (^∗∗^*p* < 0.01 between control and 5T4 GOF at Day 1 or 2, two-way ANOVA following the Bonferroni *post hoc* test; *n* = 12 areas of two independent experiments per point).

*5T4* was highly expressed in proliferating cells; therefore, we counted the number of these cells in the CVP. Six hours after injecting WT and *5T4-*KO mice with BrdU, coronal sections were prepared and subjected to IHC with an anti-BrdU antibody. BrdU^+^ cells were mainly observed in the basal part of the lingual epithelium within the CVP (Figure 5A), as previously reported (Feng et al., 2014). Remarkably, the total number of BrdU^+^ cells in the CVP was 1.5-fold higher in *5T4-*KO mice than in WT mice (WT 0.00063 ± 0.00005/μm^2^, *5T4*-KO 0.00094 ± 0.00006/μm^2^; Figure 5B). These results suggest that the number of proliferating cells such as epithelial progenitors (taste stem/progenitor cells) is higher in *5T4*-KO than in WT mice.

Consistently, stable cell lines expressing human *5T4* exhibit a reduced proliferation rate, especially in serum-free medium (Carsberg et al., 1995). To investigate whether transient expression of mouse *5T4* affects cell proliferation, we performed gain-of-function (GOF) experiments by overexpressing mouse *5T4* in HEK293T cells. To this end, HEK293T cells were transfected with p*CAG*-*H2B-EGFP* with or without p*CAG*-*5T4.* The total number of EGFP^+^ cells was lower in samples co-transfected with p*CAG*-*H2B-EGFP* and p*CAG*-*5T4* than in samples transfected with p*CAG*-*H2B-EGFP* alone (Figure 5B). Transient overexpression of *5T4* decreased proliferation of HEK293T cells, indicating that 5T4 inhibits cell proliferation. Therefore, both loss and gain of *5T4* function experiments suggested that 5T4 inhibits proliferation of taste stem/progenitor cells in the CVP.

**FIGURE 6:**
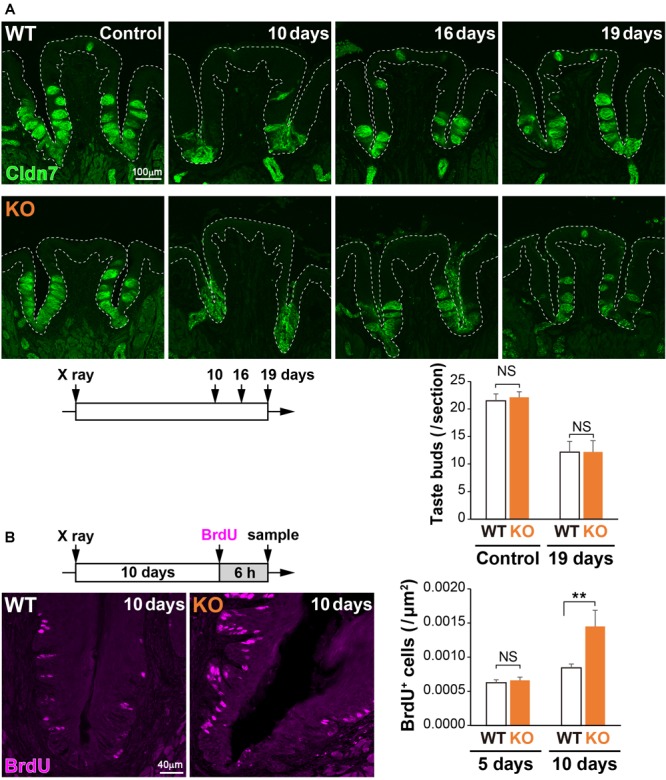
Cell proliferation in the CVP is increased more in *5T4*-KO mice than in WT mice after X-ray irradiation. (**A**, Top) The CVPs of WT and *5T4*-KO mice at 10, 16, and 19 days after X-ray irradiation were sectioned and subjected to IHC with an anti-Cldn7 antibody (a marker of TBCs; green). Images are Z-stack projections of seven sections (0.56 μm/slice). Scale bars, 100 μm. White dotted lines indicate the area of the lingual epithelium. (Bottom) The number of taste buds is expressed as the mean ± SEM (NS: not significant between WT and *5T4*-KO mice at 0 and 19 days, respectively; two-way ANOVA following the Bonferroni *post hoc* test; for control, *n* = 12 sections from six WT mice and 8 sections from four *5T4*-KO mice; for 19 days, *n* = 6 sections from three WT mice and *n* = 5 sections from three *5T4*-KO mice). (**B**, Left) Cell proliferation in the CVPs of WT and *5T4-*KO mice at 10 days after irradiation. WT and *5T4*-KO mice were intraperitoneally injected with BrdU. Six hours after BrdU injection, cells in the CVP were immunostained with an anti-BrdU antibody (magenta). Images are Z-stack projections of five sections (0.56 μm/slice). Scale bars, 40 μm. (Right) Numbers of BrdU^+^ cells are expressed as the mean ± SEM [NS, not significant (*p* = 0.7042) and ^∗∗^*p* < 0.001 between WT and *5T4*-KO mice at 5 and 10 days, respectively; two-way ANOVA following the Bonferroni *post hoc* test; for 5 days, *n* = 23 sections from eight WT mice and eight *5T4*-KO mice; for 10 days, *n* = 23 sections from eight WT mice and *n* = 8 sections from three *5T4*-KO mice].

Physical insults, such as X-ray irradiation, lead to injury followed by recovery of the lingual epithelium in the filiform papilla (Tanaka et al., 2013) or the CVP (Nguyen et al., 2012). To study the potential role of 5T4 in the injured tongue, coronal sections of WT and *5T4*-KO mice were prepared after X-ray irradiation and subjected to IHC with an anti-Cldn7 antibody. The number of Cldn7^+^ TBCs was remarkably decreased at ∼10 days after irradiation in both WT and *5T4-*KO mice, but began to increase after 16 days in order to reshape the taste buds (Figure 6A). At 19 days after insult, the number of taste buds was almost the same between WT and *5T4*-KO mice (Figure 6A). The temporal pattern of Cldn7 signal recovery did not differ significantly between WT and *5T4*-KO mice.

To further examine cell proliferation within the CVP after irradiation, WT and *5T4-*KO mice were injected with BrdU at 5 or 10 days after irradiation, and coronal sections were prepared from both groups of mice at 6 h after injection. Under normal conditions, the number of BrdU^+^ cells was higher in *5T4*-KO mice than in WT mice (Figure 5A). X-ray irradiation decreased the number of proliferating cells in the lingual epithelium; however, the number of BrdU^+^ cells in the CVP at 5 days after irradiation did not differ between WT and *5T4*-KO mice (WT 0.00063 ± 0.00004/μm^2^, *5T4*-KO 0.00066 ± 0.00005/μm^2^; Figure 6B). At 10 days after irradiation, at which point the taste buds started to regenerate, the number of BrdU^+^ cells was remarkably increased in both groups of mice, in comparison with the number at 5 days after irradiation (Figure 6B). The total number of BrdU^+^ cells in the CVP at 10 days after irradiation was increased more in *5T4*-KO mice than in WT mice (WT 0.00084 ± 0.00006/μm^2^, *5T4*-KO 0.00145 ± 0.00024/μm^2^; Figure 6B). Intriguingly, the number of proliferating cells in the CVP was higher in irradiated *5T4*-KO mice than in non-irradiated *5T4*-KO mice (Figure 6B). These results reveal that 5T4 negatively regulates proliferation of taste stem/progenitor cells in the CVP, even upon delivery of a physical insult.

## Discussion

In this study, we found that (1) *5T4* was strongly expressed in Krt5^+^ epithelial progenitor cells and Shh^+^ taste precursor cells, but weakly in mature taste receptor cells, (2) proliferating cells were more abundant in the CVP in *5T4-*KO mice than in WT mice, while cell differentiation did not differ between these mice, and (3) X-ray irradiation enhanced cell proliferation more in *5T4*-KO mice than in WT mice.

It has been reported that 5T4 is both induced by and negatively regulates a canonical Wnt/β-catenin pathway, which facilitates the response to a non-canonical pathway (Kagermeier-Schenk et al., 2011; Zhao et al., 2014; Yoshihara et al., 2016). Analyses of BATGAL reporter mice, which express *β-galactosidase* in cells with activated *β-catenin* (Maretto et al., 2003), showed that β-catenin is activated mainly in TBCs, such as Type IV, and subsets of taste receptor cells in adult mice (Gaillard and Barlow, 2011). Our results revealed that *5T4* is strongly expressed in epithelial progenitors and taste precursor cells (Figure 2), and that it may also reduce Wnt/β-catenin signaling in epithelial progenitors and control their proliferation rate (Figure 5). Consistently, Shh^+^ signals were higher in the CVP of *5T4*-KO mice (Figure 4), as reported previously in *β-catenin*-overexpressing mice (Gaillard et al., 2015).

Recent studies have shown that Lgr5 (leucine-rich repeat-containing G-protein coupled receptor 5) is a marker of distinct stem cell populations, such as those of the intestine, stomach, and skin (Barker et al., 2007, 2010; Jaks et al., 2008; Snippert et al., 2010). Within the CVP, *Lgr5* expression is strong in stem cells located at the bottom of trench area (Krt14^+^ Lgr5high) and weak in epithelial progenitor cells in the basal part of the lingual epithelium (Sox2^+^ Krt5^+^ Krt14^+^ Lgr5low) (Yee et al., 2013; Feng et al., 2014). Single *Lgr5-* or *Lgr6*-expressing cells produce all types of taste receptor cells and non-taste epithelial cells within the CVP in cultured organoids (Ren et al., 2014). Lgr5 interacts with the Wnt co-receptor LRP6 (low-density lipoprotein receptor-related protein 6) (Carmon et al., 2012), and facilitates Wnt/β-catenin signaling (de Lau et al., 2011). Interestingly, 5T4 is also known to interact with LRP5/6 to inhibit Wnt/β-catenin signaling (Kagermeier-Schenk et al., 2011). Future studies on the relationship between 5T4 and Lgr5/6 in epithelial progenitor cells should help elucidate the molecular mechanism regulating the maintenance of taste buds in the CVP.

Under healthy conditions, TBCs and neighboring epithelial cells are maintained by continuous turnover of cells. However, TBCs are injured and lost in several scenarios, which are relevant to human health (Miura and Barlow, 2010). Taste receptor cells must interact with nerve cells for their survival. Consequently, the numbers of TBCs in the fungiform papilla and CVP are reduced by transection of the chorda tympani branch of the VIIth nerve (Whitehead et al., 1987; Oliver and Whitehead, 1992; Oakley et al., 1993; Sollars et al., 2002; Oakley and Witt, 2004; Guagliardo and Hill, 2007) and the glossopharyngeal nerve of cranial ganglion IX (Wong et al., 1994; Seta et al., 1999; Miura et al., 2004), respectively. On the other hand, the mouse irradiation model mimics several key aspects of clinical radiotherapy. Head and neck cancer patients receive a series of radiotherapy sessions, which often reduce their taste sensitivity. In the current study, mice within a chamber were irradiated with 18.5 Gy of X-rays to examine how TBCs are affected chronologically (Figure 6), as described previously (Conger and Wells, 1969; Mossman et al., 1979; Nelson, 1998; Yamazaki et al., 2010; Nguyen et al., 2012). The number of proliferating progenitor cells in the basal part was remarkably reduced after irradiating either the lingual epithelium of the tongue (Tanaka et al., 2013) or the CVP (Nguyen et al., 2012). It is assumed that actively proliferating cells such as taste stem/progenitor cells (Krt5^+^ PCNA^+^ 5T4^+^) are more vulnerable to radiation-induced DNA damage than post-mitotic cells including Type I–III TBCs (PCNA^-^ 5T4^-^). Consistently, the number of BrdU^+^ newborn cells was remarkably decreased at 5 days after irradiation (Figure 6). Taste receptor cells continuously turnover at 10–14 day intervals (Beidler and Smallman, 1965; Farbman, 1980; Perea-Martinez et al., 2013). Actually, the number of taste buds was remarkably reduced at 10 days after irradiation (Figure 6). The number of proliferating cells such as taste stem/progenitor cells (Krt5^+^ PCNA^+^ 5T4^+^) in the CVP was higher in irradiated *5T4-*KO mice than in non-irradiated *5T4*-KO mice (Figure 6). However, the temporal pattern of TBC regeneration did not differ between WT and *5T4*-KO mice (Figure 6). Proliferation of taste stem/progenitor cells in *5T4-*KO mice may be insufficient to generate taste receptor cells and to facilitate reshaping of taste buds. Integration of the proliferation of taste progenitor cells with the differentiation of taste precursor cells may be necessary to augment the regeneration of taste buds in both healthy and injured *5T4-*KO mice.

## Data Availability

The datasets generated for this study are available on request to the corresponding author.

## Ethics Statement

Animal experiments were approved by the Animal Care Committee of Nara Medical University in accordance with the policies established in the NIH Guide for the Care and Use of Laboratory Animals.

## Author Contributions

YT, HT, and AT designed the study. YT and HT conducted the experiments. PS generated the *5T4*-KO mice. YT, HT, TK, and AT wrote the manuscript.

## Conflict of Interest Statement

The authors declare that the research was conducted in the absence of any commercial or financial relationships that could be construed as a potential conflict of interest.

## Supplementary Material

FIGURE S1 *5T4* is expressed in the mouse tongue. **(A,B)** ISH of the CVP of 6-week-old WT mice using the *5T4* or *Tas1r2* anti-sense RNA probe. Enlarged images of the regions enclosed by white squares in **(A,B)** are shown in (a and b). Scale bars, 100 μm in **(A)** and 50 μm in (a). **(C)** IHC of the CVPs of 6-week-old WT and *5T4*-KO mice using the antibody against 5T4 (green). Images are single confocal optical sections (2.02 μm/slice). Scale bars, 100 μm.

FIGURE S2 Levels of gene expression in CVPs from *5T4*-KO and WT mice, after normalization with *18S ribosomal* RNA. RT-qPCR analysis of total RNA extracted from WT and *5T4*-KO CVPs. **(A)** Relative amounts of *18S ribosomal* RNA were calculated using *Actb* mRNA as an internal control (NS not significant, Student’s *t* test with the Bonferroni correction; *n* = 4 per bar). **(B)** Relative amounts of several RNAs were calculated using *18S ribosomal* RNA as an internal control (^∗^*p* = 0.044 between WT and *5T4*-KO mice, Student’s *t* test with the Bonferroni correction; *n* = 4 per bar). Note that the level of *Shh* expression (a marker of taste precursor cells) calculated using *18S ribosomal* RNA as an internal control was higher in *5T4*-KO mice than in WT mice. Similar results were obtained when *Actb* mRNA was used as the internal control (Figure 4B).
