# Supplementary figures and images for: Expression of Oncofetal Antigen 5T4 in Murine Taste Papillae
Source: Front Cell Neurosci. 2019 Jul 31;13:343. doi: 10.3389/fncel.2019.00343 (PMC6685444; doi:10.3389/fncel.2019.00343)

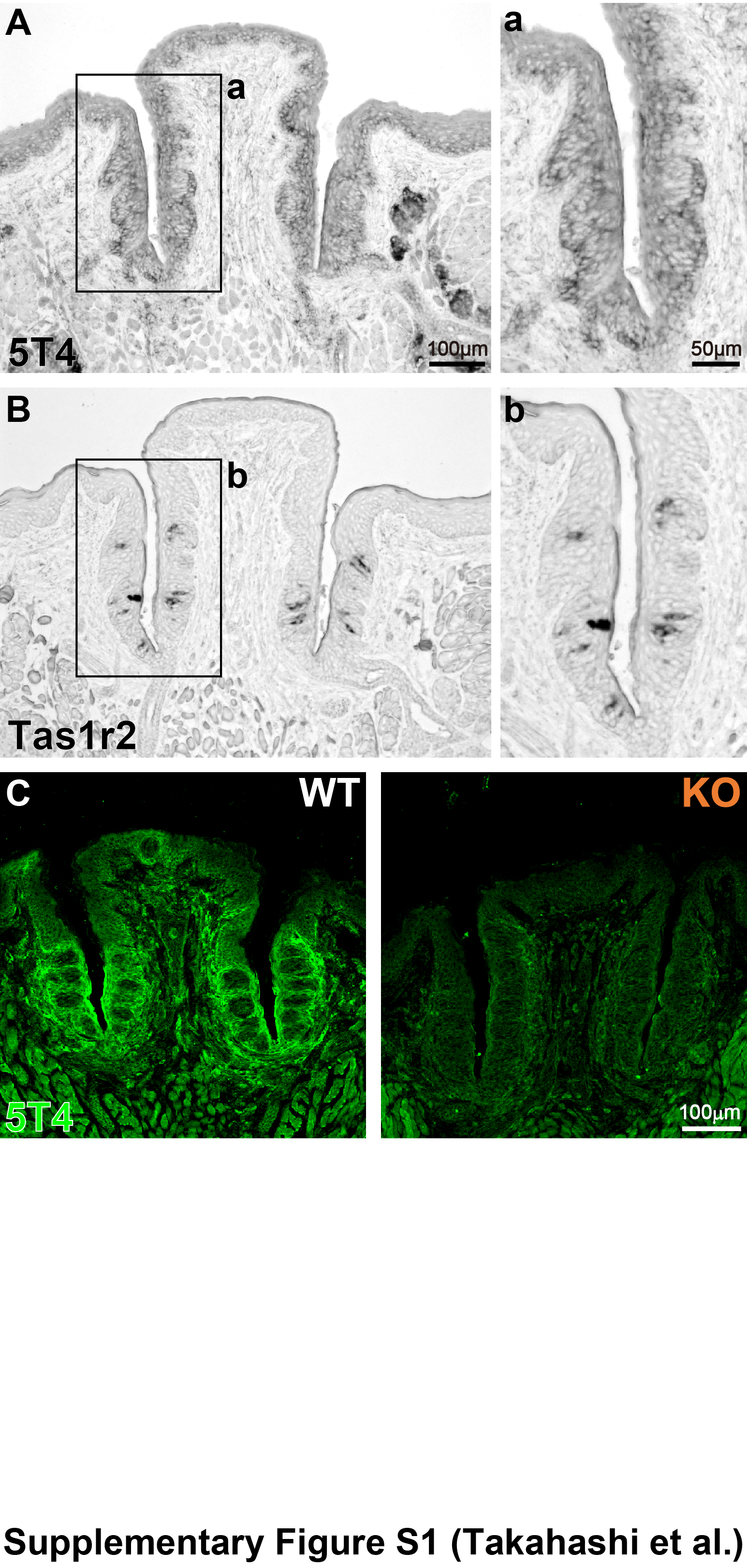

Supplement: FIGURE S1 — 5T4 is expressed in the mouse tongue. (A,B) ISH of the CVP of 6-week-old WT mice using the 5T4 or Tas1r2 anti-sense RNA probe. Enlarged images of the regions enclosed by white squares in (A,B) are shown in (a and b). Scale bars, 100 μm in (A) and 50 μm in (a). (C) IHC of the CVPs of 6-week-old WT and 5T4-KO mice using the antibody against 5T4 (green). Images are single confocal optical sections (2.02 μm/slice). Scale bars, 100 μm. [file Data_Sheet_1.ZIP › Fig.S1-6.jpg]

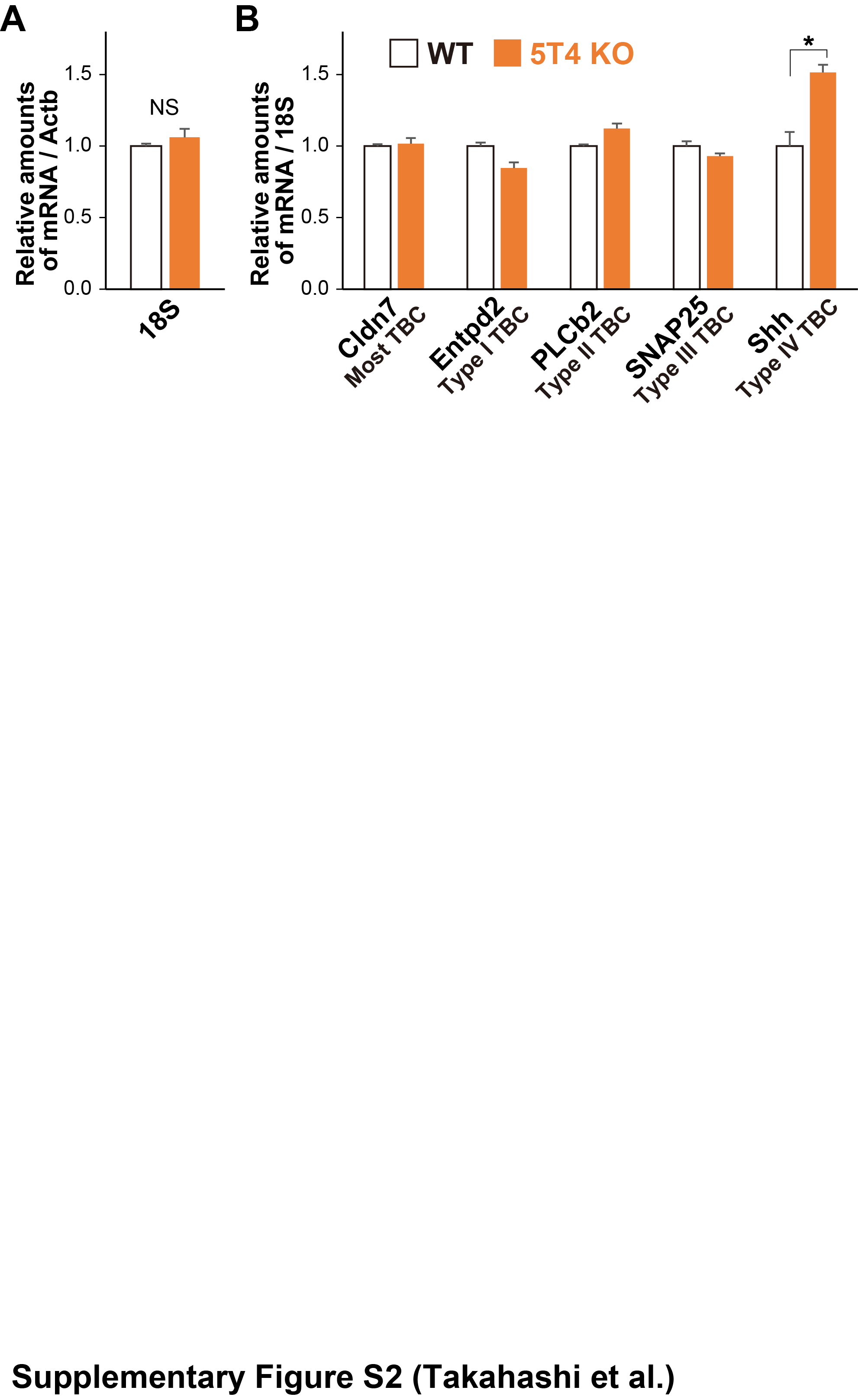

Supplement: FIGURE S1 — 5T4 is expressed in the mouse tongue. (A,B) ISH of the CVP of 6-week-old WT mice using the 5T4 or Tas1r2 anti-sense RNA probe. Enlarged images of the regions enclosed by white squares in (A,B) are shown in (a and b). Scale bars, 100 μm in (A) and 50 μm in (a). (C) IHC of the CVPs of 6-week-old WT and 5T4-KO mice using the antibody against 5T4 (green). Images are single confocal optical sections (2.02 μm/slice). Scale bars, 100 μm. [file Data_Sheet_1.ZIP › Fig.S2-2.jpg]
